# Electrocardiographic signs of cardiac ischemia at rest and during exercise in patients with COPD traveling to 3,100 m: data from a randomized trial of acetazolamide

**DOI:** 10.3389/fcvm.2025.1524201

**Published:** 2025-02-06

**Authors:** Marla Christen, Aline Buergin, Maamed Mademilov, Laura Mayer, Simon R. Schneider, Mona Lichtblau, Talant M. Sooronbaev, Silvia Ulrich, Konrad E. Bloch, Michael Furian

**Affiliations:** ^1^ Department of Respiratory Medicine, University and University Hospital of Zurich, Zurich, Switzerland; ^2^ Swiss-Kyrgyz High Altitude Medicine and Research Center, Bishkek, Kyrgyzstan; ^3^ Department of Respiratory Medicine, National Center for Cardiology and Internal Medicine, Bishkek, Kyrgyzstan

**Keywords:** aVR, cardiac ischemia, hypoxia, electrocardiogram, acetazolamide, COPD

## Abstract

**Introduction:**

In patients with chronic obstructive pulmonary disease (COPD), oxygen delivery to the heart may be impaired during travel at altitude. We assessed electrocardiogram (ECG)-derived signs of cardiac ischemia and the effects of preventive acetazolamide therapy in COPD patients traveling to high altitudes.

**Methods:**

Patients with COPD [Global Initiative for Chronic Obstructive Pulmonary Disease (GOLD) grades 2–3] and a predicted forced expiratory volume in 1 s (FEV_1_) of 66 ± 11% (mean ± SD), aged 57 ± 8 years, and living <1,000 m were included in this analysis of secondary outcomes from a randomized placebo-controlled double-blind trial (www.clinicaltrials.gov, NCT03156231). Exercise electrocardiograms were recorded at the National Center of Internal Medicine and Cardiology, Bishkek (760 m) and on the day of arrival at the Tuja Ashu high-altitude clinic (3,100 m), Kyrgyzstan. Acetazolamide (375 mg/day) or placebo was administered 24 h before the ascent and during the stay at 3,100 m. The incidence of a post-exercise ST elevation (STE) ≥0.3 mm in aVR (J + 80 ms) was the main outcome.

**Results:**

At 760 m, 3 of 49 (6%) patients randomized to placebo and 3 of 50 (6%) randomized to acetazolamide showed a post-exercise STE. At 3,100 m under placebo, two (4%) new STEs developed and one (2%) disappeared compared to 760 m (*P* = 0.564, McNemar’s test). At 3,100 m under acetazolamide, one (2%) new STE developed and two (4%) disappeared compared to 760 m (*P* = 0.564). No treatment effect was detected (*P* = 0.242, Fisher’s exact test). The mean difference (95% CI) in STE between post-peak exercise between 3,100 m and 760 m was 0.22 mm (0.06 to 0.39) and 0.09 mm (−0.06 to 0.24) under placebo and acetazolamide therapy [treatment effect, −0.13 mm (−0.35 to 0.08, *P* = 0.230)], respectively.

**Conclusions:**

In lowlanders with moderate to severe COPD ascending to 3,100 m, no ECG-derived signs of cardiac ischemia emerged neither at rest nor post-exercise and this was not modified by preventive acetazolamide therapy.

## Introduction

Chronic obstructive pulmonary disease (COPD) has a global prevalence of 8%–15%, is ranked ninth among causes of years of life lost, and is expected to rise to the fourth rank by 2040 (1). Patients with COPD suffer from ventilatory and pulmonary gas exchange impairments associated with chronic hypoxemia, which may lead to pulmonary hypertension or ischemic heart disease. The main symptoms of COPD are chronic cough, dyspnea, and exercise impairment near sea level. Due to its high prevalence in the general population, COPD is also expected to be common among mountain tourists.

At high altitudes, the reduced barometric pressure and lower inspiratory partial pressure of oxygen exacerbate hypoxemia in patients with COPD and they may promote right heart impairments and cardiac repolarization disturbances (2–7). The higher prevalence of cardiovascular disease in COPD patients further elevates their risk of cardiac ischemia in hypoxemic conditions, as suggested by previous reports (8).

At low altitudes, recent research showed that a post-exercise elevation in the ST segment in aVR of ≥0.3 mm (STE) is a robust predictor of myocardial ischemia independent of ST depressions in V5 and clinical factors (9). However, reports on electrocardiogram (ECG) changes in COPD at high altitudes are scant. They include subclinical prolongations in the QT interval (10) and reductions in the ST segment in V5 at rest and during exercise (11). Furthermore, the effects of preventive treatment with acetazolamide, a drug used to prevent acute mountain sickness (AMS) in healthy individuals, have not been studied (10, 11).

Therefore, the purpose of this study was to test the hypotheses that patients with COPD will develop signs of cardiac ischemia in the aVR when traveling to high altitude and that this may be prevented by acetazolamide.

## Methods

### Study design and setting

This trial evaluating ECG signs of cardiac ischemia was nested within a randomized placebo-controlled double-blind parallel design trial evaluating the effect of preventive acetazolamide therapy on altitude-related adverse health effects (ARAHE) in patients with COPD during a stay at high altitude (12). Cardiopulmonary exercise data have been published previously (13), however, data related to ECG and cardiac ischemia have not been previously published. The study was conducted between 1 May 2017 and 31 July 2018. Baseline evaluations were performed at the National Center of Internal Medicine and Cardiology (NCCIM, 760 m), Bishkek, Kyrgyzstan. Subsequently, patients were transferred by a minibus to the Tuja Ashu high-altitude clinic, Kyrgyzstan, located at 3,100 m. The altitude of 3,100 m represents a common but still high mountain touristic elevation to which COPD patients are able to passively travel to. Acetazolamide (375 mg/day: 125 mg in the morning and 250 mg in the evening) or similar-looking placebo capsules were orally administrated 1 day before departure and during a 2-day sojourn at 3,100 m according to a 1:1 randomization. The trial was approved by the Ethics Committee of the NCCIM (08-2016) and registered at www.clinicaltrials.gov (NCT03156231). Participants gave written informed consent.

### Participants

Men and women, aged 18–75 years, living below 800 m, diagnosed with COPD according to the GOLD guidelines (14) with a post-bronchodilator forced expiratory volume in 1 s (FEV_1_)/forced vital capacity (FVC) <0.7, predicted FEV_1_ of 40%–80%, pulse oximetry (SpO_2_) ≥92%, and partial pressure of carbon dioxide in arterial blood (PaCO_2_) <6.0 kPa at 760 m were included (*n* = 99). Exclusion criteria were COPD exacerbation, severe or unstable comorbidities, and allergy to sulfonamides.

### Assessments

Examinations included weight, height, blood pressure, heart frequency, and cardiac and pulmonary auscultation of the patients at 760 m and 3,100 m.

### Exercise testing

At 760 m before medication administration and within 4 h of arrival at 3,100 m, patients underwent cardiopulmonary exercise tests using a progressive maximal ramp protocol (15) starting at 20 W and increasing by 10 W/min to exhaustion. A four-lead ECG was continuously recorded (AMEDTEC ECGpro) and the rolling 30-s mean of the ST segment amplitude in lead aVR was computed at 80 and 60 ms from the J-point. Values from rest, peak exercise, and shortly after exercise termination (post-exercise) were extracted.

Based on a previous study providing a clinically relevant cut-off for ST segment elevation in aVR of ≥0.3 mm (9), the main outcomes of interest in this study were the altitude and acetazolamide-related STE occurrences in aVR post-exercise.

Further outcomes included exercise work rate, heart rate, blood pressure, pulse oximetry, and arterial blood gases obtained at peak exercise.

### Randomization

The participants were randomly allocated to either acetazolamide or placebo capsules by the MinimPy software (16). Drug assignment followed a 1:1 allocation ratio as per a computer-generated schedule minimizing for age (18–50 and 51–75 years), sex, and severity of airflow obstruction (predicted FEV_1_ of 40%–59% and 60%–80%). An independent pharmacist prepared active or identical placebo capsules labeled with concealed codes. The patients and researchers remained blinded until the data analysis was completed.

### Statistical analysis

All the patients randomized and included in the main project evaluating the effect of preventive acetazolamide therapy on ARAHE were included in the current trial, and therefore, no formal sample size estimation was conducted for this project (12). The statistical analysis was performed with R (Version: 4.4.0.). Based on the Shapiro–Wilk test, the data showed a normal distribution and a per-protocol analysis was performed on all available data. The results are presented as mean ± SD and mean differences (95% confidence intervals). Altitude-induced changes in the prevalence of STE ≥0.3 mm in aVR were compared using McNemar’s test, accounting for paired comparisons. The treatment effect of acetazolamide was evaluated using Fisher's exact test. A comparison was considered statistically significant with a *p*-value <0.05.

## Results

**Figure 1:**
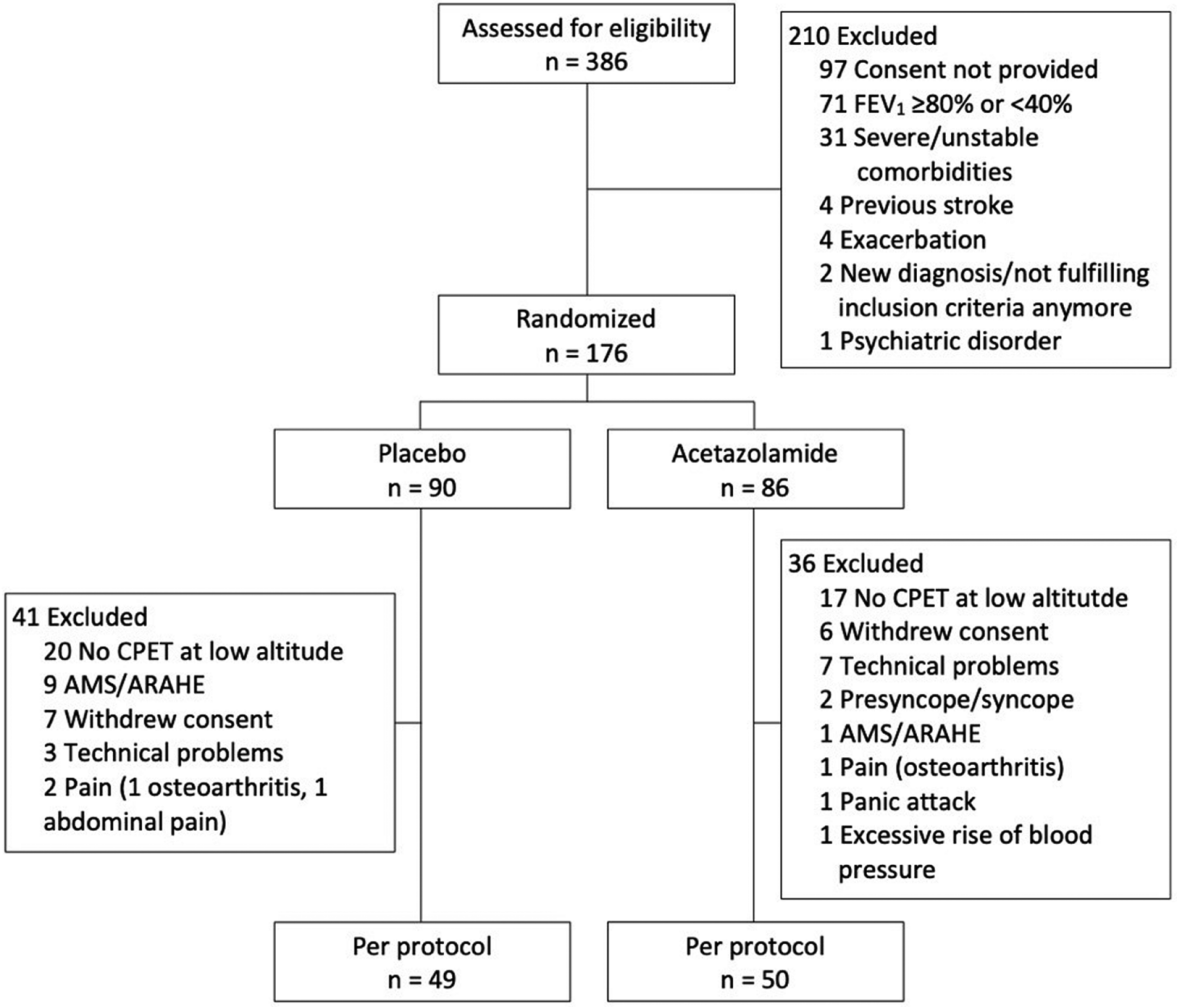
Study participant flow. AMS, acute mountain sickness; ARAHE, altitude-related adverse health effects; CPET, cardiopulmonary exercise testing; FEV_1_, forced expiratory volume in 1 s.

Of the 386 patients assessed for eligibility, 176 were randomized to either placebo (*N* = 90) or acetazolamide (*N* = 86). Of these, 77 randomized patients underwent no ECG recordings for various reasons (Figure 1). Therefore, the per-protocol analysis included the data of 49 and 50 patients randomized to placebo and acetazolamide, respectively.

The patient characteristics are presented in Table 1. The age was 57.0 ± 9.7 years (mean ± SD) for the placebo and 57.5 ± 6.6 years for the acetazolamide group. The majority of the patients (89%) were diagnosed with COPD GOLD grade 2.

**Table 1:** Patient characteristics.

Characteristic	Placebo group	Acetazolamide group
No (women)	49 (13)	50 (18)
Age, years	57.0 ± 9.7	57.5 ± 6.6
Height, m	1.64 ± 0.08	1.65 ± 0.09
Weight, kg	72.7 ± 15.2	74.4 ± 12.6
BMI, kg/m^2^	27.1 ± 5.0	27.2 ± 3.8
FEV_1_, % predicted	66 ± 12	65 ± 10
FEV_1_/FVC	0.60 ± 0.09	0.62 ± 0.08
GOLD grade (2/3)	(42/7)	(46/4)
mMRC, *n* (%)		
0	27 (55.1)	29 (58.0)
1	14 (28.6)	12 (24.0)
2	4 (8.2)	4 (8.0)
3	3 (6.1)	3 (6.0)
4	1 (2.0)	2 (4.0)
Regular medication, *n* (%)		
Oral steroid	1 (2.0)	0 (0.0)
Inhaled beta adrenergics	8 (16.3)	11 (22.0)
Inhaled anticholinergics	10 (20.4)	9 (18.0)
Inhaled corticosteroids	5 (10.2)	8 (16.0)
Antihypertensives	4 (8.2)	7 (14.0)
Aspirin	4 (8.2)	7 (14.0)

BMI, body mass index; FEV_1_, forced expiratory volume in 1 s; FVC, forced vital capacity; GOLD, Global Initiative for Chronic Obstructive Lung Disease; mMRC, modified Medical Research Council dyspnea score.

Values are presented as mean ± SD or No. (%).

### Electrocardiogram

The ST segment in lead aVR at 80 ms from the J-point (J + 80 ms) revealed a slight but statistically significant change in the placebo group when comparing post-exercise STE at 3,100 m to that at 760 m with a mean difference (95% CI) of 0.22 mm (0.06–0.39, *p* < 0.05). However, in all other measurements at J + 80 ms or J + 60 ms, there were no significant changes in the ST segment in aVR due to altitude or treatment with acetazolamide (Table 2).

**Table 2:** ST amplitudes in lead aVR and clinical evaluation.

Variable	Placebo		Δ mean difference (3,100 m − 760 m) (95% CI)	Acetazolamide		Δ mean difference (3,100 m − 760 m) (95% CI)	Treatment effect (95% CI)	*P*-value (treatment effect)
	760 m	3,100 m		760 m	3,100 m			
HR peak, bpm	135 ± 3	134 ± 3	−1 (−6 to 5)	135 ± 3	129 ± 3	−6 (−11 to 0)	−5 (−12 to 3)	0.227
Maximal load, watts	107 ± 4	97 ± 4	−11 (−14 to −7)	104 ± 4	90 ± 4	−14 (−17 to −11)	−3 (−8 to 1)	0.140
SpO_2_ peak, %	93.9 ± 0.5	84.2 ± 0.5	−9.7 (−10.5 to −9.0)	93.7 ± 0.5	85.9 ± 0.5	−7.7 (−8.5 to −7.0)	2.0 (1.0 to 3.1)	0.002
PaO_2_ peak, kPa	10.1 ± 0.1	7.6 ± 0.1	−2.5 (−2.7 to −2.3)	9.8 ± 0.1	8.1 ± 0.1	−1.7 (−1.9 to −1.6)	0.8 (0.5 to 1.1)	<0.001
PaCO_2_ peak, kPa	5.2 ± 0.1	4.8 ± 0.1	−0.4 (−0.5 to −0.3)	5.1 ± 0.1	4.4 ± 0.1	−0.7 (−0.8 to −0.6)	−0.3 (−0.5 to −0.2)	<0.001
ST in aVR with j + 60 ms, mm								
At rest	−0.35 ± 0.06	−0.30 ± 0.06	0.05 (−0.06 to 0.17)	−0.29 ± 0.06	−0.30 ± 0.06	0.00 (−0.12 to 0.12)	−0.05 (−0.22 to 0.11)	0.526
At peak	−0.18 ± 0.06	−0.10 ± 0.06	0.08 (−0.04 to 0.20)	−0.20 ± 0.06	−0.21 ± 0.06	−0.01 (−0.13 to 0.11)	−0.09 (−0.26 to 0.07)	0.273
Post-peak exercise	−0.44 ± 0.06	−0.32 ± 0.06	0.12 (0.00 to 0.25)	−0.33 ± 0.06	−0.31 ± 0.06	0.02 (−0.10 to 0.14)	−0.10 (−0.27 to 0.08)	0.269
Change from rest to peak exercise	0.17 ± 0.06	0.20 ± 0.06	0.03 (−0.13 to 0.20)	0.09 ± 0.06	0.08 ± 0.06	−0.01 (−0.17 to 0.15)	−0.04 (−0.27 to 0.19)	0.711
Change from rest to post-peak exercise	−0.09 ± 0.06	−0.02 ± 0.06	0.07 (−0.10 to 0.24)	−0.04 ± 0.06	−0.01 ± 0.06	0.03 (−0.14 to 0.19)	−0.05 (−0.28 to 0.19)	0.919
ST in aVR with j + 80 ms, mm								
At rest	−0.49 ± 0.07	−0.40 ± 0.07	0.09 (−0.05 to 0.23)	−0.41 ± 0.07	−0.38 ± 0.07	0.03 (−0.12 to 0.17)	−0.06 (−0.27 to 0.14)	0.543
At peak	−0.48 ± 0.07	−0.34 ± 0.07	0.14 (−0.01 to 0.29)	−0.54 ± 0.07	−0.41 ± 0.07	0.12 (−0.02 to 0.27)	−0.02 (−0.22 to 0.19)	0.882
Post-peak exercise	−0.79 ± 0.08	−0.57 ± 0.08	0.22 (0.06 to 0.39)	−0.69 ± 0.07	−0.60 ± 0.07	0.09 (−0.06 to 0.24)	−0.13 (−0.35 to 0.08)	0.230
Change from rest to peak exercise	0.01 (−0.14 to 0.16)	0.06 (−0.09 to 0.20)	0.05 (−0.16 to 0.25)	−0.13 (−0.27 to 0.01)	−0.03 (−0.18 to 0.11)	0.10 (−0.11 to 0.30)	0.05 (−0.24 to 0.34)	0.642
Change from rest to post-peak exercise	−0.30 (−0.46 to −0.15)	−0.17 (−0.32 to −0.02)	0.13 (−0.08 to 0.35)	−0.28 (−0.43 to −0.14)	−0.22 (−0.37 to −0.07)	0.06 (−0.14 to 0.27)	−0.07 (−0.37 to 0.23)	0.741

AZA, acetazolamide; bpm, beats per minute; CI, 95% confidence interval; HR, heart rate; SpO_2_, oxygen saturation measured by pulse oximetry; SD, standard deviation; PaCO_2_, partial pressure of carbon dioxide in arterial blood; PaO_2_, partial pressure of oxygen in arterial blood.

Values presented as mean ± SE or mean difference (95% CI).

**Figure 2:**
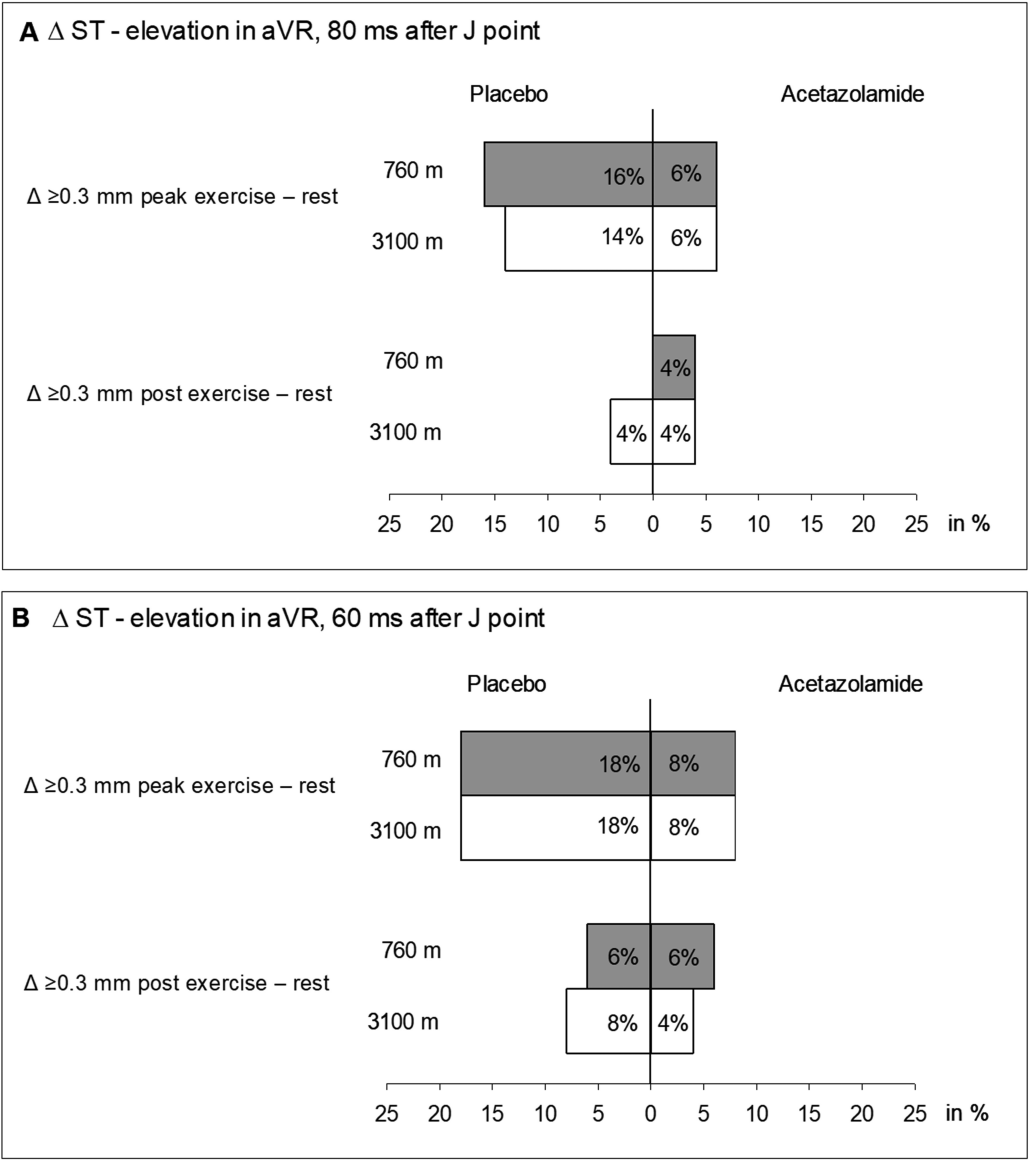
Incidence of ST segment elevations ≥0.3 ms from values at rest measured in aVR at peak exercise and post-exercise. **(A)** ST segment measured from the J-point + 80 ms. **(B)** ST segment measured from the J-point + 60 ms.

At 760 m and J + 60, we observed that three (6%) patients assigned to placebo and three (6%) patients assigned to acetazolamide had an STE of ≥0.3 mm in aVR post-peak exercise (Figure 2B). At J + 80, none and two (4%) of the patients assigned to placebo and acetazolamide, respectively, exceeded the STE threshold of ≥0.3 mm at 760 m (Figure 2A) post-peak exercise. Post-peak exercise at 3,100 m under placebo and J + 60, two (4%) new STEs ≥0.3 mm appeared and 1 (2%) STE disappeared compared to 760 m (*P* = 0.564, McNemar’s test). With acetazolamide, one (2%) new STE ≥0.3 mm appeared and two (4%) disappeared compared to 760 m (*P* = 0.564, McNemar’s test). No treatment effect was detected (*P* = 0.242, Fisher’s exact test). Correspondingly, at J + 80 under placebo, two (4%) new STEs were observed (*P* = 0.157, McNemar’s test); under acetazolamide, one (2%) new STE and one (2%) absent STE was observed compared to 760 m (*P* = 1.000 McNemar’s test). No effect of acetazolamide treatment was detected (*P* = 0.242, Fisher’s exact test). The results were confirmed when comparing STEs at either J + 60 or J + 80 at 3,100 m vs. 760 m.

In addition, no changes in the occurrence or absence of STEs during rest or peak exercise between the altitudes and interventions were observed.

### Cardiopulmonary exercise tests

The maximal work rate (Wmax) was significantly reduced at 3,100 m compared to 760 m in both groups by a mean difference (95% CI) of 11 W (7–14) and 14 W (11–17) under placebo and acetazolamide, respectively (Table 2). Acetazolamide showed no significant treatment effect regarding Wmax in comparison to placebo. Arterial blood gas analysis at peak exercise revealed significant changes with the ascent from 760 to 3,100 m, i.e., a mean difference of −9.7% for SpO_2_ (−10.5 to −9.0), partial pressure of oxygen in arterial blood (PaO_2_) of −2.5 kPa (−2.7 to −2.3), and PaCO_2_ of −0.4 kPa (−0.5 to −0.3). Acetazolamide significantly mitigated altitude-related decrements in SpO_2_ and PaO_2_ at peak exercise by 2.0% (1.0–3.1) and 0.8 kPa (0.5–1.1), respectively, whereas PaCO_2_ was further reduced by −0.3 kPa (−0.5 to −0.2) (Table 2).

In total, 68 (76%) of 90 patients randomized to placebo experienced various ARAHE during the stay at 3,100 m while the incidence of ARAHE in the acetazolamide group was 42 of 86 (49%, *p* < 0.001, chi-squared test) (12).

## Discussion

We analyzed ST segment changes in aVR as signs of cardiac ischemia at rest, at peak, and post-peak exercise in patients with COPD during a sojourn at 3,100 m and treated with either placebo or acetazolamide. We found that approximately 6% of the lowlanders with moderate to severe COPD did have clinically relevant STEs at 760 m, but no further worsening during the first hours at 3,100 m was observed. This percentage was similar in patients with COPD ascending to 3,100 m under acetazolamide prophylaxis. Although the patients with COPD experienced various ARAHE while at 3,100 m that were partially prevented by acetazolamide (12), no clinically relevant ECG abnormalities were found to be associated.

Data on ECG changes upon exposure to altitude in the general population and patients with COPD are scant. Only two related studies by our research group were identified in a detailed search of the literature (10, 11). The study by Bisang et al. reported prolonged QT intervals corrected for heart rate in patients with COPD staying at 2,048 m, which were not prevented by nocturnal oxygen therapy (10). ST changes were not reported. In a previous study by our group, we conducted 12-lead ECGs in patients with moderate to severe COPD who traveled to 3,100 m and exercised after the same time spent at the target altitude (11). This study confirms our findings that altitude exposure did not induce ST segment changes in V5. It is reassuring that neither this study nor the cited study by Carta et al. found new STEs at rest or peak exercise at 3,100 m.

Apart from confirming the findings of Carta et al., this study reports ECG data post-exercise for the first time. Notably, STE in aVR post-exercise had the highest prognostic value for predicting myocardial ischemia in a previous study by Wagener et al. (9). In the study, 1,596 patients in whom exercise-inducible cardiac ischemia was suspected, were examined. ST segment amplitudes in leads aVR and V5 were measured automatically. As a reference as to whether the patient actually had ischemia or not, myocardial perfusion-single photon emission computed tomography (MP-SPECT) was used. Maximal accuracy (area under the curve = 0.62) was achieved in lead aVR with a cut-off value of 0.030 mV (equal to 0.3 mm) for STE at 2 min into recovery and 80 ms after the J-point. The results further showed that in patients with cardiac ischemia, the STE was significantly higher than in non-ischemic patients in leads aVR and V5. The diagnostic accuracy for ischemia of lead aVR alone was equally as high as lead V5.

Accordingly, due to the simplicity and worldwide feasibility of applying a four-lead ECG, we decided in our study to focus exclusively on lead aVR and chose the same cut-off value of 0.3 mm. Regardless of whether the measurements were taken at J + 60 or J + 80 ms, our study was not able to show a significant change in ST segments due to altitude or acetazolamide. Although the statistically significant increase in STE (at J + 60 and J + 80 ms) from rest to post-exercise at 3,100 m compared to 760 m under placebo suggested provocation of some degree of cardiac ischemia, the clinical relevance of this finding remains to be elucidated.

Our study is the first to estimate the prevalence of STE in patients with COPD at rest and during exercise at low and high altitudes. McKinney et al. (17) tried to determine the prevalence of STE in aVR in a population of over 2,000 patients referred for exercise ECG recordings for evaluation of the diagnosis of coronary artery disease. They automatically measured the STE at 80 ms after the J-point and used a cut-off value of STE ≥1 mm. All STEs above 0.75 mm were additionally checked by a cardiologist, which may have increased the accuracy of this study. Their results showed that 3.4% of the patients had an STE in aVR. In the current study, we found low proportions of post-exercise STE of 0% and 6% at J + 60 and J + 80 ms, respectively (Figure 2).

Our data revealed a significant decrease in work capacity when comparing lowland to high-altitude conditions. This decline can primarily be attributed to the combined effects of reduced oxygen availability, cardiovascular adaptations, and metabolic changes, which collectively contribute to the observed reduction in maximal work capacity at high altitudes. Indeed, a decrease in SpO_2_, PaO_2_, and PaCO_2_ at peak exercise at high altitudes has previously been observed in patients with COPD (18, 19). Our findings also confirm the results of Jonk et al. (20). They observed blood gas changes in healthy individuals during exercise in normoxic and hypoxic conditions under placebo and acetazolamide treatment. The hyperventilation induced by acetazolamide is consistent with the improvements in SpO_2_ and PaO_2_, however, acetazolamide did not improve exercise performance. Indeed, the effect of acetazolamide on maximal exercise performance at high altitudes remains under debate and needs further investigation (21).

The current study investigated STE changes in non-severely hypoxemic and stable patients with COPD without serious or unstable cardiopulmonary comorbidities. Therefore, this population is representative of a large community of patients with COPD but the results should not be extrapolated to patients with more severe COPD with associated hypoxemia or hypercapnia at low altitudes.

The focus of the current study was to investigate STE changes in aVR, which can be assessed by a simple four-lead ECG recording. However, this limitation restricts interpretation as ST changes in other regions of the heart could not be detected and it was not possible to compare ST changes to other leads. Moreover, since the exercise testing was conducted on the first day at 3,100 m and only the second day of medication administration, it is possible that this study did not detect further differences between the two groups that may have become apparent over a longer period of time.

In addition, more participants in the placebo group were excluded due to AMS. As AMS symptoms can be partially attributed to pronounced hypoxemia, this may have introduced bias into our results since pathophysiological ischemic signs are more likely to be expected in individuals experiencing greater hypoxemia.

## Conclusion

This study investigated STE changes in non-severely hypoxemic and stable patients with COPD in a high-altitude environment (3,100 m). The findings indicate that while STE in aVR did not show significant changes due to high altitude or preventive acetazolamide therapy, there was a slight increase in STE post-exercise at high compared to low altitudes, suggesting a potential risk for cardiac ischemia. These results highlight the need for further studies to understand the clinical significance of these ECG changes in patients with COPD and to explore preventive strategies for those at risk when traveling to high altitudes.

## Data Availability

The raw data supporting the conclusions of this article will be made available by the authors upon reasonable request.

## References

[1] Foreman KJ Marquez N Dolgert A Fukutaki K Fullman N McGaughey M Forecasting life expectancy, years of life lost, and all-cause and cause-specific mortality for 250 causes of death: reference and alternative scenarios for 2016–40 for 195 countries and territories. Lancet. (2018) 392(10159):2052–90. 10.1016/S0140-6736(18)31694-5 30340847 PMC6227505

[2] Furian M Lichtblau M Aeschbacher SS Estebesova B Emilov B Sheraliev U Efficacy of dexamethasone in preventing acute mountain sickness in COPD patients: randomized trial. Chest. (2018) 154(4):788–97. 10.1016/j.chest.2018.06.006 29909285

[3] Furian M Lichtblau M Aeschbacher SS Estebesova B Emilov B Sheraliev U Effect of dexamethasone on nocturnal oxygenation in lowlanders with chronic obstructive pulmonary disease traveling to 3100 meters: a randomized clinical trial. JAMA Netw Open. (2019) 2(2):e190067. 10.1001/jamanetworkopen.2019.0067 30794302 PMC6484579

[4] Tan L Latshang TD Aeschbacher SS Huber F Flueck D Lichtblau M Effect of nocturnal oxygen therapy on nocturnal hypoxemia and sleep apnea among patients with chronic obstructive pulmonary disease traveling to 2048 meters: a randomized clinical trial. JAMA Netw Open. (2020) 3(6):e207940. 10.1001/jamanetworkopen.2020.7940 32568400 PMC7309443

[5] Latshang TD Tardent RPM Furian M Flueck D Segitz SD Mueller-Mottet S Sleep and breathing disturbances in patients with chronic obstructive pulmonary disease traveling to altitude: a randomized trial. Sleep. (2018) 42:1–10. 10.1093/sleep/zsy203 30517695

[6] Lichtblau M Furian M Aeschbacher SS Bisang M Ulrich S Saxer S Dexamethasone improves pulmonary hemodynamics in COPD-patients going to altitude: a randomized trial. Int J Cardiol. (2019) 283:159–64. 10.1016/j.ijcard.2018.12.052 30638985

[7] Lichtblau M Latshang TD Furian M Muller-Mottet S Kuest S Tanner F Right and left heart function in lowlanders with COPD at altitude: data from a randomized study. Respiration. (2019) 97(2):125–34. 10.1159/000492898 30269143

[8] Mallet RT Burtscher J Richalet JP Millet GP Burtscher M. Impact of high altitude on cardiovascular health: current perspectives. Vasc Health Risk Manag. (2021) 17:317–35. 10.2147/VHRM.S294121 34135590 PMC8197622

[9] Wagener M Abächerli R Honegger U Schaerli N Prêtre G Twerenbold R Diagnostic and prognostic value of lead aVR during exercise testing in patients suspected of having myocardial ischemia. Am J Cardiol. (2017) 119(7):959–66. 10.1016/j.amjcard.2016.11.056 28215415

[10] Bisang M Latshang TD Aeschbacher SS Huber F Flueck D Lichtblau M Nocturnal heart rate and cardiac repolarization in lowlanders with chronic obstructive pulmonary disease at high altitude: data from a randomized. Placebo-controlled trial of nocturnal oxygen therapy. Front Med. (2021) 8(129):557369. 10.3389/fmed.2021.557369 PMC7956979 33732710

[11] Carta AF Bitos K Furian M Mademilov M Sheraliev U Marazhapov NH ECG changes at rest and during exercise in lowlanders with COPD travelling to 3100 m. Int J Cardiol. (2021) 324:173–9. 10.1016/j.ijcard.2020.09.055 32987054

[12] Furian M Mademilov M Buergin A Scheiwiller Philipp M Mayer L Schneider S Acetazolamide to prevent adverse altitude effects in COPD and healthy adults. NEJM Evid. (2022) 1(1):EVIDoa2100006. 10.1056/EVIDoa2100006 38296630

[13] Kind RF Furian M Buergin A Scheiwiller PM Mayer L Schneider SR Effects of acetazolamide on exercise performance in patients with COPD going to high altitude: randomised controlled trial. ERJ Open Res. (2024) 11(1):00767-2024. 10.1183/23120541.00767-2024 PMC11744325 39834599

[14] Global Strategy for the Diagnosis, Management, and Prevention of Chronic Obstructive Pulmonary Disease. 2020 Report. (2020). Available online at: https://goldcopd.org/gold-reports/ (Accessed August 14, 2019).

[15] Ross RM. ATS/ACCP statement on cardiopulmonary exercise testing. Am J Respir Crit Care Med. (2003) 167(10):1451. 10.1164/ajrccm.167.10.950 12738602

[16] Saghaei M. An overview of randomization and minimization programs for randomized clinical trials. J Med Signals Sens. (2011) 1(1):55–61. 10.4103/2228-7477.83520 22606659 PMC3317766

[17] McKinney J Pitcher I Fordyce CB Yousefi M Yeo TJ Ignaszewski A Prevalence and associated clinical characteristics of exercise-induced ST-segment elevation in lead aVR. PLoS One. (2016) 11(7):e0160185. 10.1371/journal.pone.0160185 27467388 PMC4965008

[18] Furian M Flueck D Latshang TD Scheiwiller PM Segitz SD Mueller-Mottet S Exercise performance and symptoms in lowlanders with COPD ascending to moderate altitude: randomized trial. Int J Chron Obstruct Pulmon Dis. (2018) 13:3529–38. 10.2147/COPD.S173039 30464436 PMC6208550

[19] Furian M Hartmann SE Latshang TD Flueck D Murer C Scheiwiller PM Exercise performance of lowlanders with COPD at 2,590 m: data from a randomized trial. Respiration. (2018) 95(6):422–32. 10.1159/000486450 29502125

[20] Jonk AM Van Den Berg IP Olfert IM Wray DW Arai T Hopkins SR Effect of acetazolamide on pulmonary and muscle gas exchange during normoxic and hypoxic exercise. J Physiol (Lond). (2007) 579(3):909–21. 10.1113/jphysiol.2006.120949 17218362 PMC2151360

[21] Kind R Furian M Buergin A Scheiwiller P Mayer L Schneider S Effects of acetazolamide on exercise performance in patients with COPD at high altitude. RCT. Eur Respir J. (2019) 54(suppl 63):PA1631. 10.1183/23120541.00767-2024 PMC11744325 39834599

